# The Epidemiology of Fatal road traffic Collisions in Trinidad and Tobago, West Indies (2000–2011)

**DOI:** 10.3402/gha.v9.32518

**Published:** 2016-11-09

**Authors:** Chavin D. Gopaul, Aruna Singh-Gopaul, Joan M. Sutherland, Luke Rostant, Kristie L. Ebi, Dave D. Chadee

**Affiliations:** 1 Department of Para-Clinical Sciences, Faculty of Medicine, University of the West Indies, St. Augustine, Trinidad, West Indies; 2 North West Regional Health Authority, St. George Central, Barataria, Trinidad; 3 Department of Life Sciences, University of the West Indies, St. Augustine, Trinidad, West Indies; 4 Department of Global Health, School of Public Health, University of Washington, Seattle, WA, USA

**Keywords:** road traffic collisions, fatality, time, place, person, Trinidad, West Indies

## Abstract

Summary of what was known

• Few studies on road traffic collisions conducted in Latin American and the Caribbean region

• Increasing car ownership within the region

• Lack of seatbelt usage in Trinidad

• Most accidents are associated with alcohol usage

• High mortality among pedestrians

Summary of what this study adds

• Most collisions occur at night

• Most collisions occur at weekends

• Collisions are evenly distributed throughout the year

• More males than females die in fatal collisions

• The most productive age group (15–44) is affected the most

• More drivers than pedestrians are affected

• Reflects the pattern of developed rather than developing countries with respect to time of day for RTCs

**Background:**

The purpose of the study is to determine the epidemiology of road traffic collisions (RTCs) in Trinidad and Tobago by characterizing RTCs in terms of number of collisions, fatalities, victim profiles, and locations for the purpose of informing accident prevention programs. Previous studies of RTCs in Trinidad and Tobago were primarily concerned with patterns of drivers use of seat belts, road collisions as a cause of mortality in young men, and the economic burden of road collisions. Attempts were made to model road fatalities, but limited epidemiological data meant that it was difficult to determine trends or develop models.

**Methods:**

This study determined the epidemiology of RTCs in Trinidad and Tobago over the period 2000–2011 using data collected by the Trinidad and Tobago Road Traffic Branch of the Police Service and secondary data from the Central Statistical Office. Data were analyzed using Excel, SPSS, and R statistical packages.

**Results:**

Fatalities were greater among men (80%) than among women (20%) and were highest on two major freeways in Trinidad [the Churchill–Roosevelt Highway and the Sir Solomon Hochoy Highway]. Most collisions occurred during the night among individuals between the ages of 15 and 44 years. Fatalities among drivers steadily increased over the study period and overtook fatalities among pedestrians, who were the group most affected in 2000. Most fatalities occurred at weekends.

**Conclusions:**

These patterns can inform (i) education programs and (ii) road and traffic control measures.

## Introduction

Worldwide, road traffic accidents/collisions (RTAs or RTCs) account for over 1.2 million deaths per year, with the majority occurring in low- and middle-income countries (1, 2). RTCs increase in low-income countries as gross domestic product (GDP) increases (2). GDP is a sign of increased standard of living and productivity, which are the characteristics of developing third world countries. Trinidad and Tobago is a small twin island in the Caribbean, which is a third world country that is an economic hub in the region. It experienced a boost in the vehicle industry in 1995 and the opening of the foreign-used car market, allowing for consumers to improve their standard of living by buying a car and contribute to a higher GDP. The costs associated with deaths and long-term disability for accident survivors and their families stress low-income countries. Health and motor insurance companies in these low-income countries cannot fully absorb the costs incurred from collisions and injuries that may have been sustained by the insured. Thus, the insurance industry and markets struggle to compete and survive in this economic environment. The insurance industry and the premiums paid by the insured absorb these costs in high-income countries (3). Factors responsible for causing RTCs worldwide regardless of state-of-the-art highways and infrastructure include environmental conditions, speed, loss of control, and ignoring road safety (4, 5); use of mobile phones (5, 6); unsafe road surfaces (7); traffic volume and road rage (8); and drunk driving, work stress, inexperience, and failure to look properly (4, 5, 9).

In the Region of the Americas, RTCs are the leading cause of death in children aged 5–14 years and caused over 142,000 deaths in 2007 according to the Pan American Health Organization (10). Motor vehicle collisions have a 25% mortality rate in the region, with pedestrians the common victims (1). In the region of the Americas, the distribution of vehicles reported in collisions ranged cars (55.5%), buses (1.9%), heavy trucks (6.9%), motor bikes (3.6%), and other categories of vehicles as defined by traffic regulatory agencies (34.1%) (10). Studies conducted by Barreto et al. (11), Holder (12), St. Bernard and Mathews (7), and Ramroop et al. (13) provide some of the evidence required for launching studies on RTC prevention in the Caribbean region by the World Health Organization (WHO).

The Republic of Trinidad and Tobago is in dire need for traffic regulatory bodies and agencies as there is none that can be held liable for road safety and collision prevention (1). Each year road fatalities continue to rise and the carnage on the nation's roadways is left unaddressed as there are no collision prevention programs in place. Responsibility for coordinating road safety, education, and accident prevention is carried out by non-government organizations (NGOs) and the Ministry of Health. RTCs continue to be an incessant issue faced by all users of the roadways and legislating bodies. These factors have played crucial roles in the purpose of this study.

In the past decade, traffic authorities in Trinidad and Tobago attempted to reduce the incidence of RTCs by public service announcements as educational programs advising on the benefits of wearing seat belts. Road transport is considered the most crucial means for moving goods and people within the Twin Islands, and so alternative means of transport are not an option (13). The demand for privately owned cars has increased in the past two decades. To meet the demand for vehicles, in 1995, the government allowed the importation of second-hand Japanese vehicles. This led to significantly increased numbers of vehicles on the road. This further contributed to traffic congestion on major roads and increased the load for agencies to properly manage the volume of vehicles with little infrastructure and regulations (11). Furthermore, the number of cases of driving while under the influence of alcohol has increased, as have speeding and reckless driving (4, 5, 10).

The aim of this study is to determine the epidemiology of RTCs in Trinidad and Tobago by characterizing RTCs in terms of numbers of crashes, fatalities, victim profiles, and locations, for the purpose of informing accident prevention programs.

## Methods

### Study area

Trinidad and Tobago are twin sister islands located in the southernmost part of the Caribbean archipelago (14). Trinidad has an estimated population of 1.3 million inhabitants (15), with one-third of the island designated as cultivable land (15) and the remaining dedicated to roadways, highways, housing, and commercial enterprises. The country is politically stable, and economic growth depends mainly on its petrochemical industry. It is considered an economic hub for other Caribbean countries and is considered a key leader in the CARICOM and regional Commonwealth countries. The country thus has developing infrastructure and open trade with Asia, the Americas, and Europe. This growing economy facilitates a foreign-used car industry, which, in turns, contributes to the country's economy and GDP. The business environment also depends heavily on road transport for trade and delivery of goods and services.

The island experiences a tropical climate with average daily temperatures ranging between 22 and 32°C (16). Trinidad has two distinct seasons: a dry season from December to May and a rainy season from May to November (16). The climate varies from very hot to very rainy, with road transport playing a pivotal role in the movement of people, goods, and services under a range of environmental conditions.

### Data sources

#### Data from the Traffic and Highway Patrol Unit, Trinidad and Tobago Police Services

Data for the period 2000–2011 were obtained from the Traffic and Highway Patrol Unit on the number of fatal vehicle collisions and the number of fatalities by gender, age, class of road user, day of the week, time of the day, and the road where the collision occurred.

#### Data from the Central Statistical Office of Trinidad and Tobago

Data for the period 2000–2011 were obtained from the Central Statistical Office of Trinidad and Tobago and cross-referenced with that from the Traffic and Highway Patrol Unit in order to check its accuracy. Differences with the statistical representations from the Central Statistics Office and from the Trinidad and Tobago Road Traffic Branch could not be resolved because of missing data.

### Data analysis

Raw data were entered into SPSS (Statistics Desktop Version 22.0 Win 32 FP1 Ref#4037142, 2014) (17) for analysis, and charts were generated using Excel. Road fatality data were explored in R (R core team 2012) (18) with respect to gender, time of day, day of the week, age, class, location, and month using parametric statistical tests (student's *t*-test for gender and one way ANOVA for other factors) and non-parametric alternative statistical tests when parametric assumptions were not met (Mann–Whitney *U* test for the effect of gender and Kruskal–Wallis for other factors). Results from the statistical tests were compared with boxplots to visually determine which category might be responsible for any observed differences.

## Results

### General

During the period 2000–2011, the number of persons died from all causes in Trinidad Tobago was 119,020. Of these, RTCs accounted for 2,360 deaths (≈2.0%) in 2,073 fatal collisions (≈1.1 deaths per collision). The annual number of fatalities caused by RTCs averaged 196.6, ranging from a low of 126 (or 1.3%) in 2000 to a high of 233 (or 2.6%) in 2008. The year-to-year variation in the number of fatal collisions was 5.6–10.4% (Table 1). Over 56% of the collisions (1,170) and 56% of the fatalities (1,323) occurred during the period 2004–2009.

**Table 1:** The number of all-cause deaths, number of accidents and fatalities that occurred from 2000 to 2011 in Trinidad and Tobago

	All-cause mortality		Fatal road traffic accidents		Road traffic fatalities	
			
Year	No	%	No	%	No	%
2000	9,474	0.7	126	5.6	131	1.4
2001	9,736	0.7	144	6.4	160	1.6
2002	9,778	0.8	148	6.7	163	1.7
2003	10,206	0.8	167	7.5	197	1.9
2004	9,872	0.8	173	7.7	196	1.9
2005	9,885	0.8	196	8.8	216	2.2
2006	9,664	0.7	188	8.4	214	2.2
2007	9,653	0.8	188	8.4	214	2.2
2008	10,000	0.8	233	10.4	261	2.6
2009	10,200	0.8	192	8.6	222	2.1
2010	10,251	0.8	168	7.6	205	2.0
2011	10,301	0.8	150	6.7	181	1.8
Total	119,020	0.9	2,073	100	2,360	1.9

Total Trinidad and Tobago population estimated to be 1,225,225 (2011).

Source: Trinidad and Tobago traffic branch.

Monthly data were available for 1,513 fatalities from 2005 to 2011. There was considerable variability between years but no statistically significant differences in monthly fatalities (Fig. 3).

### Gender and age

**Fig. 1:**
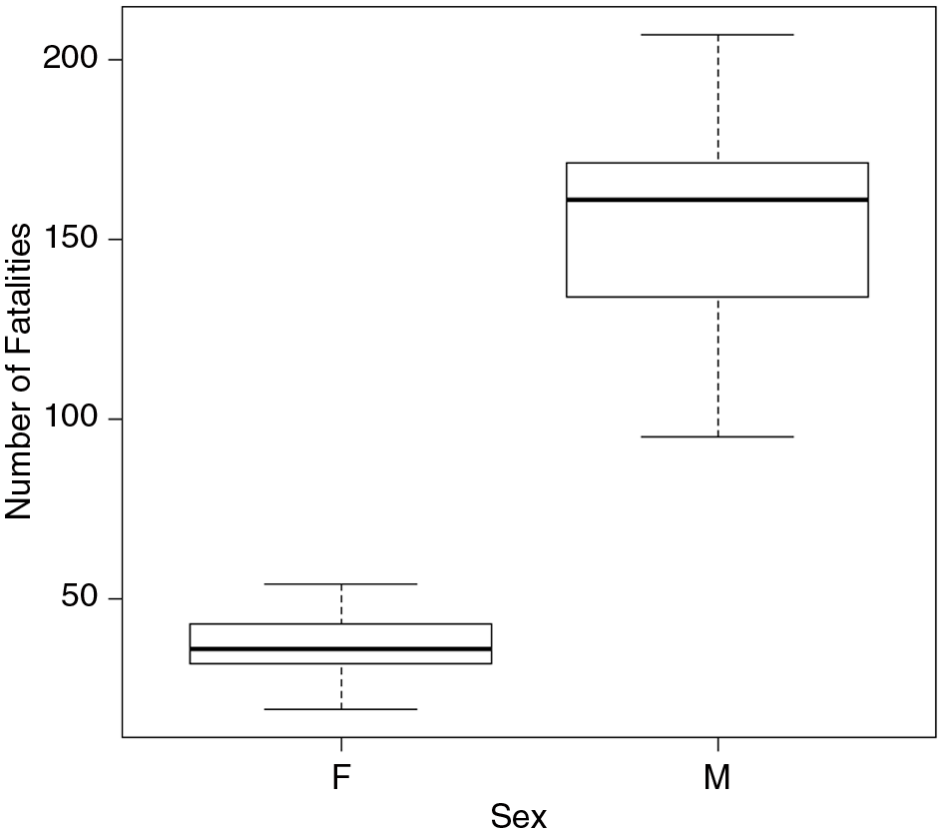
Boxplot of road fatalities by gender from 2000 to 2011.

**Fig. 2:**
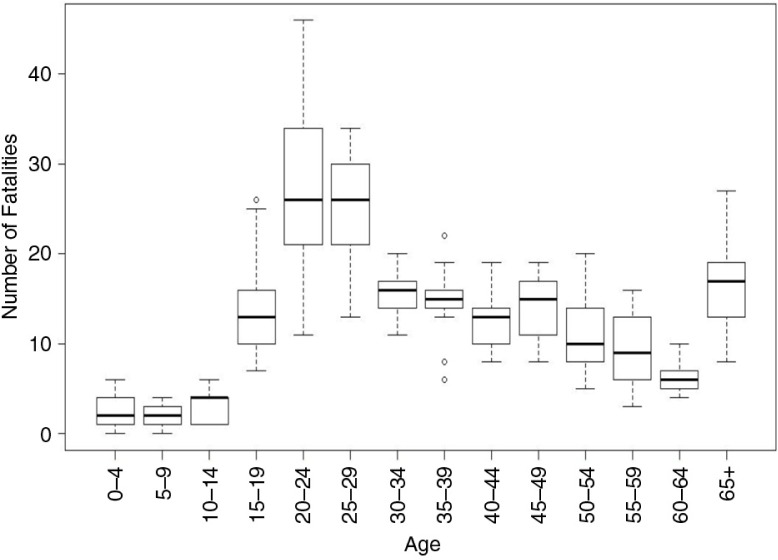
Boxplot of road fatalities by age from 2000 to 2011.

Of the 2,360 road fatalities, 80% of the fatalities (1,213) were men and 20% (299) were women, as shown in Fig. 1. Figure 2 shows fatalities by age. A significant (*p*<0.04) number of fatalities occurred in the age group of 15–29 (G=29.3; d.f.=4; *p*<0.04 for males and G=23.29; d.f.=4; *p*<0.05 for females). Over 50% of fatalities occurred in two age groups 15–29 (33.1%) and 30–44 (19.4%). The lowest number of fatalities occurred among the 0–14 and >60 age groups, which represented 3.5 and 11.2% of the population, respectively. A generally decreasing trend in the number of fatalities among the 15–29 age group occurred during the study (Table 2), with the decline in the >60 age group being significant (*p*<0.03).

**Fig. 3:**
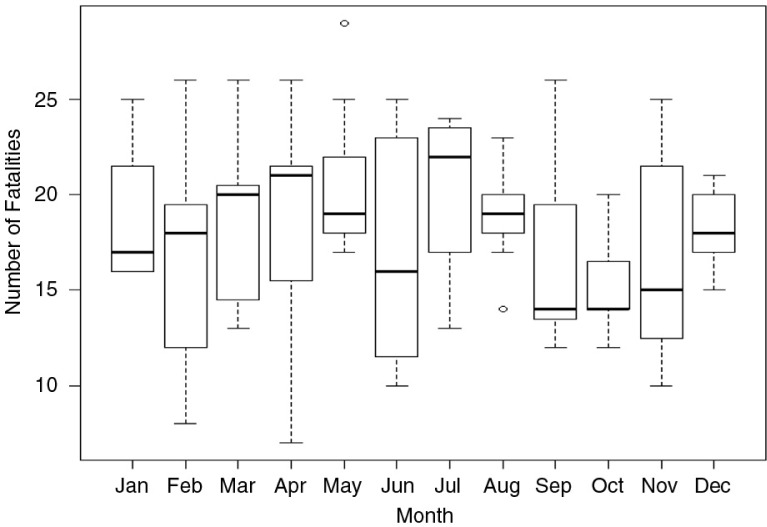
Boxplot of road traffic fatalities in Trinidad and Tobago by month (data from 2005 to 2011). Source: Trinidad and Tobago traffic branch.

**Table 2:** The gender and age groups of road traffic fatalities in Trinidad (2005–2011)

	Male		Females		Total	
			
Age categories	No	%	No	%	No	%
0–14	32	2.6	22	7.3	54	3.5
15–29	391	32.3	108	36.1	499	33.1
30–44	245	20.2	48	16.1	293	19.4
50–59	232	19.1	48	16.1	280	18.5
>60	130	10.7	40	13.4	170	11.2
Unknown	183	15.1	33	11.0	216	14.3
Total	1,213	100.0	299	100.0	1,512	100.0

Source: Trinidad and Tobago traffic branch.

### Road fatalities by day of the week

**Fig. 4:**
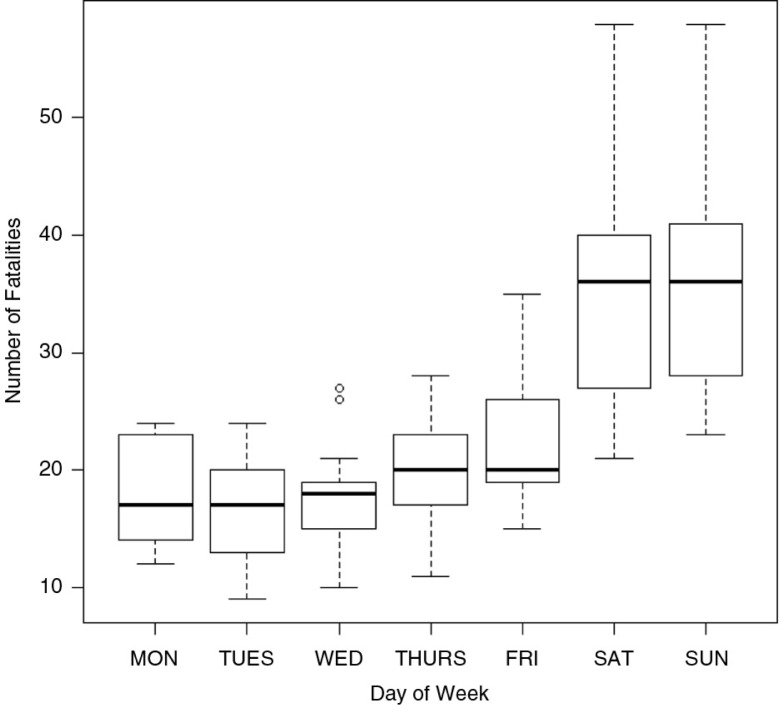
Boxplot of road traffic fatalities in Trinidad and Tobago by day of the week (data from 2000 to 2011).

**Fig. 5:**
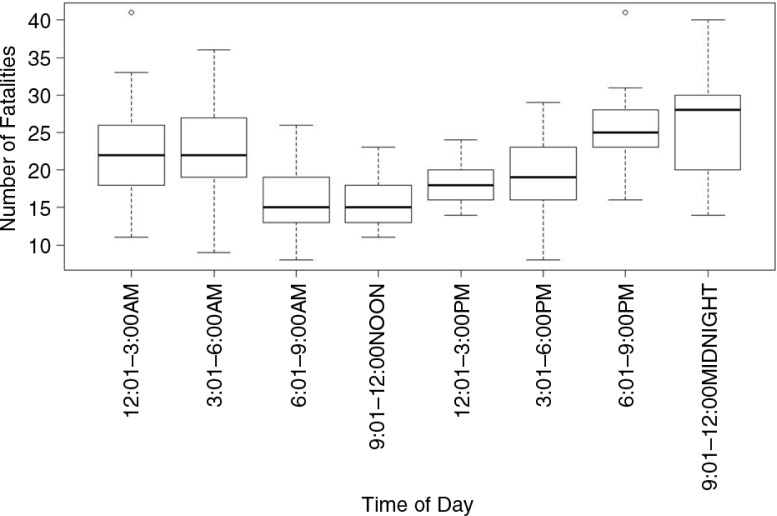
Boxplot of road traffic fatalities in Trinidad and Tobago by time of day (data from 2000 to 2011).

There were 2,167 road fatalities between 2001 and 2011. Road collisions and road fatalities occurred every day of the week in Trinidad (Fig. 4). There were significant differences between the number of fatalities based on the day of the week (Kruskal–Wallis χ^2^=49; d.f.=6; *p*<0.01). The majority of the fatalities occurred on Fridays (290 or 13.4% with a mean of 22.3 fatalities), Saturdays (455 or 21% with a mean of 35 fatalities), and Sundays (477 or 22% with a mean of 37 fatalities). The number of fatalities that occurred Monday to Thursday consistently ranged between 10 and 12% of all fatalities (Fig. 5); there were no significant differences found among fatalities from Monday to Thursday (χ^2^=2.89. d.f.=3, *p*=0.41).

### Road fatalities by time of day

The Trinidad and Tobago Traffic Branch database contained information on the time of day of the collision for 64.1% of the road fatalities (1,512 fatalities; Fig. 5). There was one significant peak (G=45.0; d.f.=7; *p*<0.03) starting from dusk at 6.00 pm, reaching a peak between 21.00 and 24.00 h (G=21.8; d.f.=7; *p*<0.03), and declining significantly (G=23.5; d.f.=7; *p*<0.04) at dawn. The results showed that most fatalities (846, 56%) occurred during twilight and nocturnal hours. Of all road fatalities, 44% occurred during daylight hours (06–18 h) with the periods 6:00 am–9 am and 3:00 pm–6:00 pm accounting for the greatest number of fatalities, 353 and 313, respectively. These periods include transitions from day to night and night to day, with varying light intensities (Fig. 5). The box plots of the number of fatalities and the time of the day showed significantly (χ^2^=33.56; d.f.=7; *p*<0.002) more fatalities occurred between 6.00 pm and 06.00 am than at other periods.

### Class of road users and collisions

**Fig. 6:**
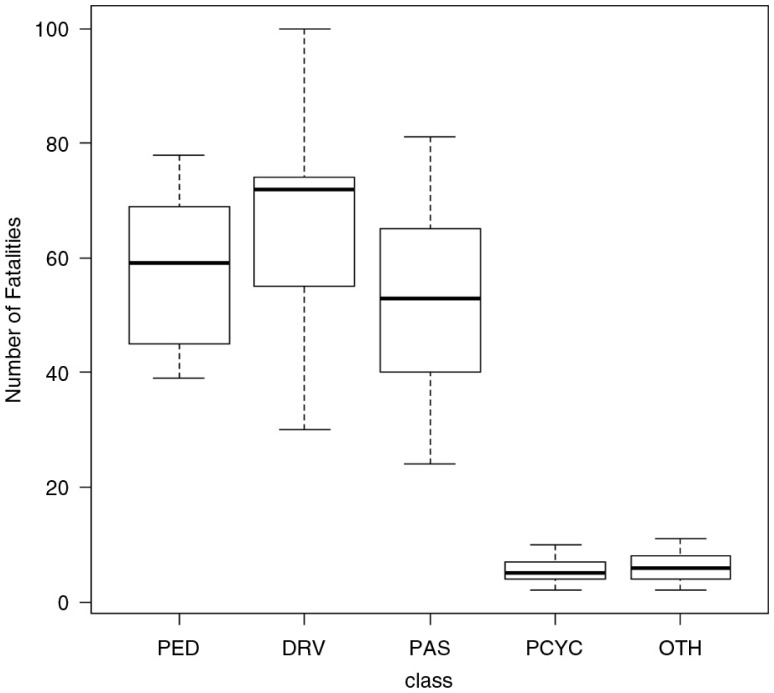
Boxplot of road traffic fatalities in Trinidad and Tobago by class (data from 2000 to 2011). PED, Pedestrian; DRV, Driver; PAS, Passenger; PCYC, Motor bikes; OTH, Others.

Records were available for 1,512 accident fatalities. There was a significant difference (Kruskal–Wallis χ^2^=48.088, d.f.=4; *p*<0.01) between road user groups (Fig. 6). Vehicle drivers accounted for 37% of fatalities (562 collisions), with 508 (90.4%) of the collisions involving males and 58 (10.3%) involving females. Passengers in vehicles accounted for 29% of fatalities (438 collisions), with males and females involved in 276 and 165 collisions, respectively. The third highest category was pedestrians, who accounted for 28% of fatalities (419 collisions), with 339 males and 80 females involved. Motorcyclists and others accounted for 6% of fatalities (90 collisions), all of which involved men.

### Roads with highest fatalities

**Fig. 7:**
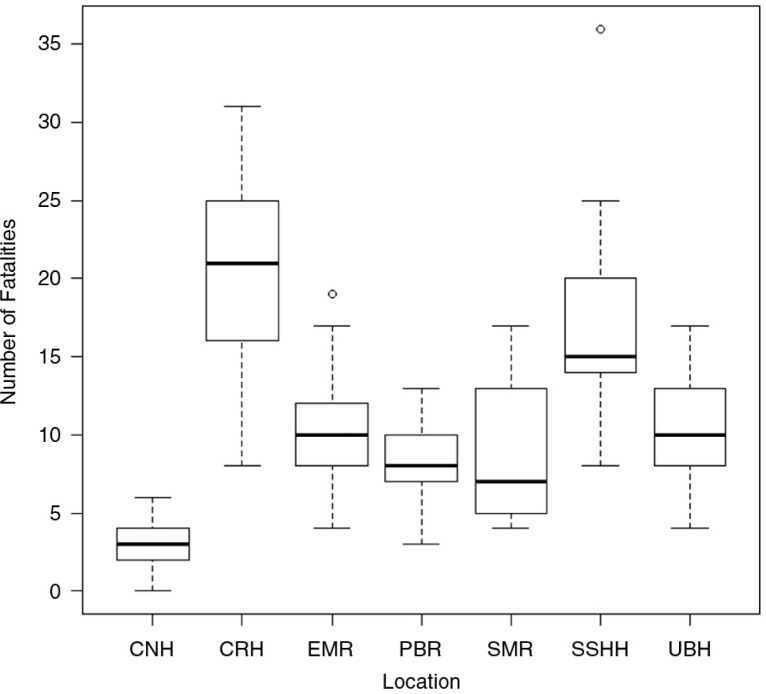
Number of fatalities that occurred on different roads in Trinidad and Tobago (from 2000 to 2011). CNH, Claude Noel Highway; SSHH, Sir Solomon Hochoy Highway; UBH, Uriah Butler Highway; SMR, Southern Main Road; PBR, Priority Bus Route; EMR, Eastern Main Road; CRH, Churchill–Roosevelt Highway.

Road fatalities varied significantly depending on location (Kruskal–Wallis χ^2^=55.01; d.f.=6; *p*<0.01) (Fig. 7). The highest average fatalities occurred on the Churchill–Roosevelt Highway (CRH), followed by the Sir Solomon Hochoy Highway (SSHH) and the Eastern Main Road. For CRH, the average number of annual fatalities was 21 (range 8–31). The CRH and the SSHH accounted for the highest incidences of fatalities, with 25 and 22%, respectively, over the analysis period.

### Profile of victims

#### Drivers

Significantly more males (80%) than females (20%) were (*p*>0.002) victims of collisions, with males aged between 15–29 and 30–44 years (G=24.6; d.f.=5; *p*<0.03). The majority (G=21.8; d.f.=7; *p*<0.03) of the collisions occurred between midnight and 06.00 am and from 6.00 pm to 12.00 am, especially during the weekends (Friday, Saturday, and Sunday).

#### Passengers

Significantly more males (G=21.3; d.f.=2; *p*>0.01) than females were involved in fatal collisions, with the majority between 15 and 29 years (*p*>0.05). The majority of the collisions occurred between midnight and 6.00 am and from 6.00 pm to 12.00 am, especially during Saturdays and Sundays.

#### Pedestrians

A significant (*p*>0.002) number of male pedestrians were killed, with the majority within the ages of 30–59 years. Most of the collisions occurred between 3:00 pm and 9.00 pm on Saturdays and Sundays.

## Discussion

The study identified the time, place, and person associated with RTCs in Trinidad and Tobago. Previous studies conducted in Trinidad and Tobago were primarily concerned with the patterns of seat belt used by drivers (19), mortality of young male drivers (12), trends in road crashes and their economic burden (7), and road fatality models (20, 21). Without robust epidemiological data, it is difficult to provide an evidence base to inform policies to effectively reduce RTCs. It should be noted that the data used in these analyses contain gaps that limit our ability to conduct time series analyses to monitor trends.

Overall, there were 2,073 fatal collisions in Trinidad and Tobago with an average annual fatality occurrence of 196.6 (range 126–233) during the period 2000–2011. Almost equal numbers of fatal collisions occurred during the traditional wet (May–December) and dry seasons (January–May). Collision patterns observed in Trinidad and Tobago peaked typically during festivals celebrated by the country, such as Christmas (December), Carnival (February), and Independence Day (August). St Bernard and Mathews (7) and Tanaboriboon and Satiennam (22) suggested heavy alcohol consumption may impair drivers’ judgment. The results demonstrated a peak in RTCs on the weekends, from Friday to Sundays. Similarly, Odero et al. (23) had reviewed all the published and unpublished reports on RTAs in developing countries from 1966 to May 1994 and reported that in 79% of the studies collisions occurred between Friday and Monday (24). However, earlier studies in Trinidad (13) showed a uniform pattern of injuries during Tuesday to Friday, which was seen to be 206 injuries on Tuesday and 275 on a Friday between 200 and 2011. There was a slight increase in the number of injuries during the weekend (Saturday and Sunday). This study found significant (*p*<0.03) numbers of fatalities occurring during the period Friday to Sunday with fewer collisions occurring from Monday to Thursday. Studies conducted in other regions of the world showed similar results with over 50% of the weekly traffic collisions and injuries occurring on Friday, Saturday, and Sunday, with a peak on Saturday (23, 25). In Trinidad, the peak occurred on Sunday, which is likely associated with alcohol consumption. The pattern of weekend crashes has long been associated with alcohol consumption or drink-driving in industrialized countries (23, 24, 26), but similar patterns are emerging in developing countries such as Papua New Guinea (27) and Trinidad and Tobago (this study). The observed pattern (13) may be the result of the study design and the data collection point being the Accident and Emergency Unit rather than specific RTCs.

With respect to the time of day most collisions occurred, our analyses suggest that most collisions occurred during the night. This differs from Odero et al. (23), who reported that 60–80% of road traffic causalities occurred during the day and one-third occurred during the night, particularly between 6:00 pm and 12:00 am. This study showed an increase starting at dusk, continuing throughout the night, and ending at dawn. Light intensities vary during dawn and dusk; persons with nyctalopia or night blindness (which slows the adjustment or switching of the rods and cones within the eyes) are at greater risk of being involved in collisions during these periods. The majority of pedestrian collisions occurred between 3.00 pm and 9:00 pm on Saturdays and Sundays, suggesting that pedestrians may not have been able to judge the distance of the oncoming traffic because of age, vision impairment (nyctalopia), or driver factor such as speeding, or a combination of all three factors. In addition, shift workers may be at risk of falling asleep while driving and this condition can be made worse if under the influence of alcohol (9). Interestingly, other studies suggest that the high incidence of day time fatalities could be explained by a greater volume of traffic on roads during the day when people travel to work, children travel to school, and most business and manufacturing centers are open for transactions. The decline in road fatalities at night in other studies may be due to lower traffic volume at night and fewer night-time activities and travel. There is some evidence that in developing countries, road fatalities occur primarily during the day while in developed and industrialized countries, most collisions occur during the night (23, 28). Therefore, the current Trinidad and Tobago road traffic data set reflects more of a developed industrialized country pattern (7, 11–13, 19).

This study showed that the majority of RTCs occurred on the CRH (25%) and the SSHH (23%) between 6:00 pm and 6:00 am on Fridays, Saturdays, and Sundays, which largely limits the age group exposed during this ‘high-risk period’. These two highways are the two larger of the four highways and cover greater distances connecting northern to southern and eastern to western parts of Trinidad. These two highways experience heavy vehicular traffic during the week, and drivers are forced to adhere to speed limits. However, during the weekends, there is less traffic and this facilitates speeding and greater chances of road collisions especially of under the influence of alcohol or drugs. Therefore, it is not surprising that the most significant age group affected by RTCs was the 15–29 and 30–44 years age groups as people within these age groups are more likely to indulge in entertainment activities during these hours over the weekend. Like Trinidad, Jha and Agrawal (29) observed that Sundays had the highest number of vehicular collisions in cities in India. This suggests most of the fatalities are unlikely to be work related, but may be associated with drinking and driving as people engage in recreational activities over the weekend. The high proportion of fatalities that occurred between the hours of 9:00 pm and 12:00 am suggests that these motorists were homebound, post-recreational activity.

The high fatality count found on the CRH and SSHH is consistent with that described by St Bernard and Mathews (7), in which RTCs were more common on urban than rural roads. Similar results were found in India (30), where 122,000 road fatalities were recorded for 2001–2003 on highways in New Delhi.

In Peru, Thailand, and mostly pedestrians accounted for over 80% of the road traffic deaths (11, 23). Analyses of fatalities based on the class of road user indicated that the number of fatalities among drivers has been increasing and overtook pedestrians, who were leading in the year 2000. Therefore, it is not surprising that the results showed a large number of road fatalities occurred among drivers and passengers, with the majority of the deaths recorded among males (80%).

An analysis based on age of the road accident victims showed that people aged 15–29 and 30–44 years constituted the highest proportion of the fatalities. People in these age groups represent the working population. A review of motor vehicle fatalities in Canada (31) found that deaths among teens and young adults (<30 years) accounted for the greatest number (17.3%) of deaths. Similar results were reported in Japan and Ireland (32). The implication is that road safety messages should be designed to target people in this age group.

## Conclusions

In conclusion, analyses of trends in fatal collisions and fatalities showed significant differences in the time of day when collisions occurred, the day of the week when most collisions occurred, the age groups most affected, the gender of victims, and roads with significantly higher fatality counts (SSHH and CRH) and with more motor vehicle drivers than pedestrians being killed. There were no statistically significant differences in fatal collisions or fatalities by month.

There is a potential opportunity to work with the Trinidad and Tobago Road Traffic Branch to establish a database using available technology such as a geographic information system to record the time and location of collisions with photographic and other evidence. While the National Road Safety Council is the lead agency for the regulation and implementation of preventative strategies with regard to collisions on the nation's highways, there is still lack of an overall national road safety strategy (33). The Ministry of Transport for Trinidad and Tobago should consider ensuring dedication of resources to the development and implementation of such a strategy, as it is the first step to address the road carnage caused by RTCs. Agencies and regulatory bodies can assert authority and conduct research and development initiatives to improve the alarming statistics surrounding the increase in road fatalities over the study period. This will include having an overall vision on road safety for the islands, engaging multiple stakeholders, and ensuring that there are measurable short-, mid-, and long-term objectives such as fatality reduction targets, as suggested by WHO (33). Research and development will evidently lead to education programs as well as road traffic control measures, given the trends and patterns we found. Findings in the study such as time of day, day of the week, and highways with the most RTCs can be used to guide road traffic control measures and regulations, while variables such as gender and age group can be used to guide educational programs geared toward reducing the number of RTCs.

Legislation is also a vital area for addressing the factors that contribute to fatalities from RTCs, such as lack of seat belt use, drink-driving, lack of helmet use, violation of speed limit, and use of mobile phones while driving. While laws for the regulation of these factors do exist in the country, the enforcement is often lacking (33). The Global Status Report on Road Safety (2015) reveals that enforcement of speed limits is ranked as number 4 on a scale of 1–10, and drink-driving is ranked as number 5 contributing cause. However, enforcement of seat belt use and helmet use are ranked higher, as numbers 7 and 9, respectively. This speaks of the need for continued advocacy to ensure that high levels of enforcement are always placed as a priority on the political agenda.

Safety of roads and vehicles is also an important priority. In Trinidad and Tobago, standards for vehicles to protect occupants and pedestrians are also lacking (33). Again, the lack of enforcement is an area that requires addressing and policies regarding these issues require development. Areas such as road design and regular inspections of existing road infrastructure also require further consideration and are important elements needed in the overall prevention of road traffic collision fatalities strategy.

RTCs can be caused not only by human error but also from conditions and activities impeding cognitive functions, especially the ability to make clear decisions and judgments. There are variables that can impact the amount of road fatalities but are not confined to time, day, roads, and its characteristics and usage, as well as age and gender. Regardless of this, RTCs continue to remain a major contributor to mortality statistics across the globe.

Ethics approval was obtained from the South West Regional Health Authority Scientific Research Ethics Committee, San Fernando, Trinidad, West Indies.

## Paper context

RTC in Trinidad and Tobago continue to plague the countries development. This study solidifies the point that a public health approach is required to deal with road traffic collisions in Trinidad and Tobago. Previous studies in Trinidad indicated that mortality was highest among pedestrians. Findings from this study demonstrate that most fatal collisions occurred at night and on weekends and that mortality among drivers was highest. There is without a doubt that stiffer penalties and more enforcement is needed to curtail this rise in deaths associated with road traffic collisions.
